# Co-design of clinician-facing report and implementation pathway for a digital questionnaire for reporting head and neck cancer symptoms

**DOI:** 10.1093/jamiaopen/ooaf130

**Published:** 2025-11-12

**Authors:** Chinasa Odo, Nikki Rousseau, Joanne Patterson, Vinidh Paleri, Theofano Tikka, Clare Schilling, Rebecca Randell

**Affiliations:** Centre for Digital Innovations in Health & Social Care, University of Bradford, Bradford BD7 1DP, United Kingdom; Wolfson Centre for Applied Health Research, Bradford BD9 6RJ, United Kingdom; Leeds Institute of Clinical Trials Research, University of Leeds, Leeds LS2 9NL, United Kingdom; University of Liverpool, Liverpool L3 5TR, United Kingdom; The Royal Marsden NHS Foundation Trust, London SW3 6JJ, United Kingdom; St George’s University Hospitals NHS Foundation Trust, London SW17 0QT, United Kingdom; University College London Hospitals NHS Foundation Trust, London NW1 2PG, United Kingdom; Centre for Digital Innovations in Health & Social Care, University of Bradford, Bradford BD7 1DP, United Kingdom; Wolfson Centre for Applied Health Research, Bradford BD9 6RJ, United Kingdom

**Keywords:** clinician, digital questionnaire, implementation, hospital, clinical report

## Abstract

**Objective:**

This research aimed to design and evaluate a clinician-facing report generated from the SYmptom iNput Clinical (SYNC) system, a digital questionnaire for patients to report Head and Neck Cancer (HNC) symptoms and uses this to calculate a risk score. The research explored how the SYNC system could be integrated into existing hospital workflows.

**Materials and Methods:**

The research comprised two studies conducted across three hospital settings. Study 1 focused on design and evaluation of the clinician-facing report, while Study 2 explored pathways for integrating the SYNC system into clinical workflows. Data collection involved six focus groups, two conducted in each hospital setting with clinicians and administrative staff involved in cancer management, to capture diverse perspectives on both the report and integration feasibility.

**Results:**

Study 1 participants emphasised the need for clear and concise presentation of critical information in the report, yet there were also differing perspectives on what constituted clear and concise presentation. Study 2 participants suggested that effective integration of the SYNC system into hospital workflows would depend on ensuring that the system is user-friendly and adaptable, to help maintain clinician engagement and support better patient management.

**Conclusions:**

Engaging a range of stakeholders, not just the end user, is crucial to gaining an understanding of the patient pathway and implementation challenges that may appear at each point, supporting the development of digital tools that are both functional and well-integrated into clinical workflows. This approach enhances usability and ensures the SYNC system can support the management of HNC patients.

## Introduction

Head and neck cancer (HNC) symptoms are often discovered by primary care practitioners (PCP) or dentists during routine examinations or when patients present related or unrelated symptoms.^1^ When there are suspected HNC symptoms, patients are referred to the hospital for further diagnosis and treatment. A study across two tertiary care centres in the United States highlighted barriers to timely HNC referrals, including delays in primary care appointments, limited PCP awareness of symptoms, and patient fear worsened by inconsistent communication of the symptoms the patients reported.^2^ One solution for addressing this communication inconsistency is timely transfer of accurate information about relevant symptoms to HNC specialists. Patients are in the best position to communicate their symptoms whereas clinicians are in the best position to contextualise the information in terms of disease continuum.^3^ In our previous study,^4^ we developed a digital technology named SYmptom iNput Clinical (SYNC), which supports patients to report HNC symptoms by completing a digital questionnaire and supports clinicians’ decision-making via an algorithm that uses the symptoms to calculate a HNC risk score. This builds on the Excel version of Head and Neck Cancer Risk Calculator (HaNCRC v2) with 77% prediction accuracy^5^ which allows the clinician to identify those patients at high risk of HNC who require urgent attention. The SYNC system also presents a dashboard to track patents journey when filling the form.

The objective of this research was to collaborate with clinicians and administrative staff across three different hospitals to develop, validate and evaluate the reports generated by the SYNC system questionnaire. Additionally, the research explored how the SYNC system could be integrated into the existing hospital workflow.

## Materials and methods

A co-design approach was employed, and data collection was conducted using focus groups across three distinct hospitals involved in HNC management. Focus groups were chosen because they facilitate the inclusion of diverse professionals from various disciplines, allowing exploration of different perspectives.^6^ The research was guided by the Technology Acceptance Model (TAM).^7^ According to TAM, a user’s intention to use technology is directly influenced by two major components: perceived usefulness (PU) and perceived ease of use (PEOU), such that for digital technology to be successfully integrated into healthcare systems and demonstrate clinical effectiveness, it must enable end-users to engage with it effortlessly.^7^

The research was divided into two distinct studies:

- Study 1 focused on co-design and evaluation of the clinician-facing report.
- Study 2 explored the implementation pathway for the SYNC system questionnaire.

### Participants

The hospitals were chosen to represent variation in service delivery models and patient characteristics in terms of the symptoms they present, which was crucial for capturing a broad range of perspectives. This diversity is essential to the validity and reliability of the findings. Purposive sampling was employed to select participants from sites A, B and C. This method was considered appropriate as it allows matching of the study samples to the objectives of the research, enhancing the trustworthiness of the data and results.^8^ Participants were selected with the assistance of research nurses at each site to ensure diversity in participants’ job roles (see Table 1 and 2). In Study 1, participants were clinicians of varying grades who were involved in the diagnosis of HNC. Study 2 included both clinical staff as well as administrative personnel. The number of participants for each focus group in Study 1 ranged between 5 and 6 (total n = 17); for Study 2, between 5 and 9 (total n = 19). These numbers were considered appropriate based on Krueger’s recommendation that the ideal size for focus groups discussing non-commercial topics is five to eight participants.^9^

**Table 1.** Study 1—clinician-facing sessions.

Focus group	ENT specialist		Maxillofacial		Consultants	Registrar	Total
	Male	Female	Male	Female			
Site A	4	–	1	–	3	2	5
Site B	2	3	1	–	6		6
Site C	1	5	–	–	3	3	6

**Table 2.** Study 2—implementation pathway sessions.

Focus group	Clinicians		Cancer service managers		Waiting list trackers		Business managers		IT officers		Total	
	Male	Female	Male	Female	Male	Female	Male	Female	Male	Female	Male	Female
Site A	1	2	–	–	–	2	2	–	1	1	4	5
Site B	1	–	1	1	–	2					2	3
Site C	1			1	1	2					2	3

### Data collection

An in-person focus group was adopted to allow more engaging interactions, particularly with clinicians who often face distractions during online sessions due to clinical duties. Six separate focus groups were conducted across Site A, B, and C, with two sessions held at each location. Each session was coordinated by two researchers, one serving as the moderator and the other as a note-taker. For both Study 1 and 2, focus group discussions were audio-recorded and transcribed verbatim.

**Figure 1.**
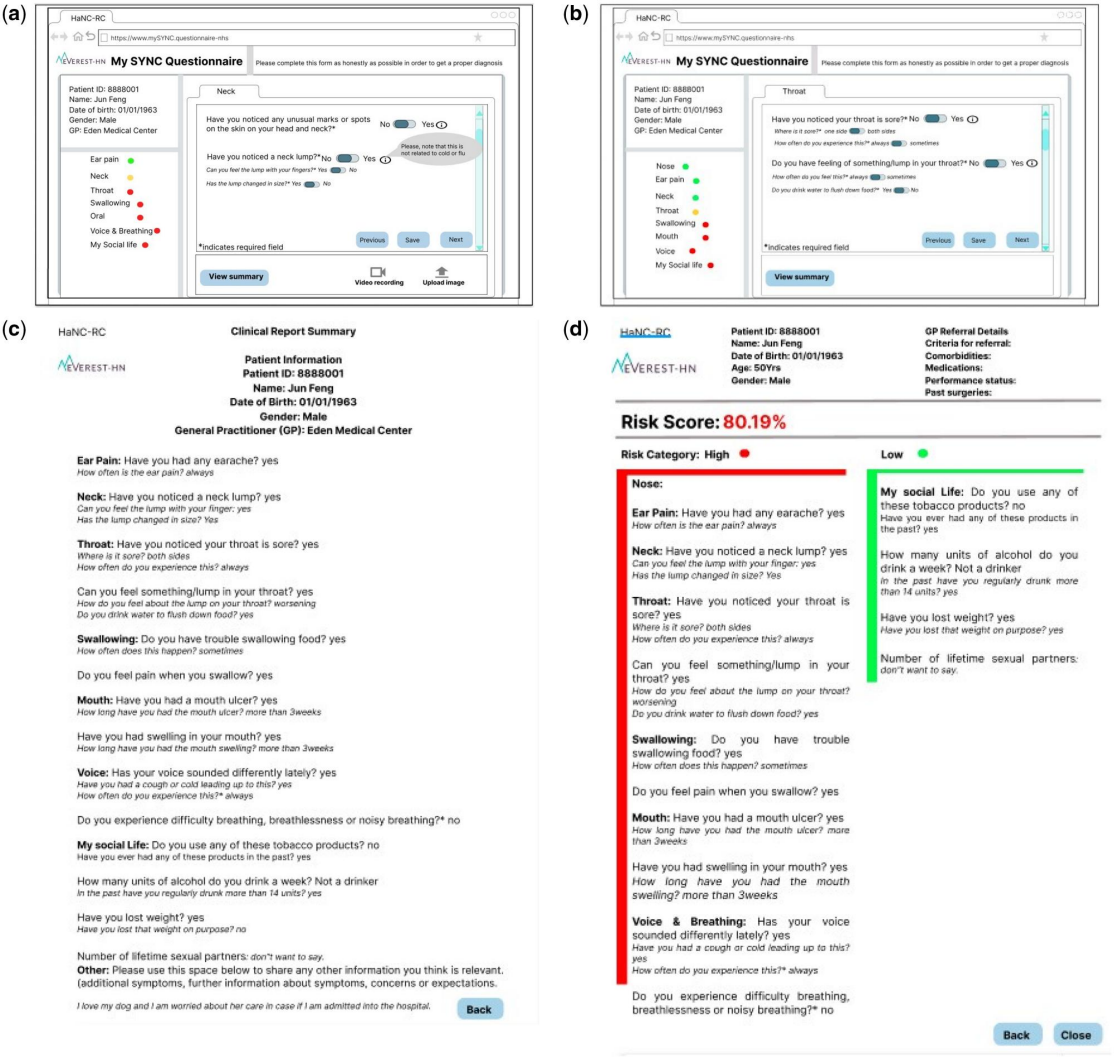
Examples of low-fidelity prototype of patient facing summary page and clinicians sketch idea created from initial workshop with clinicians.

**Figure 2.**
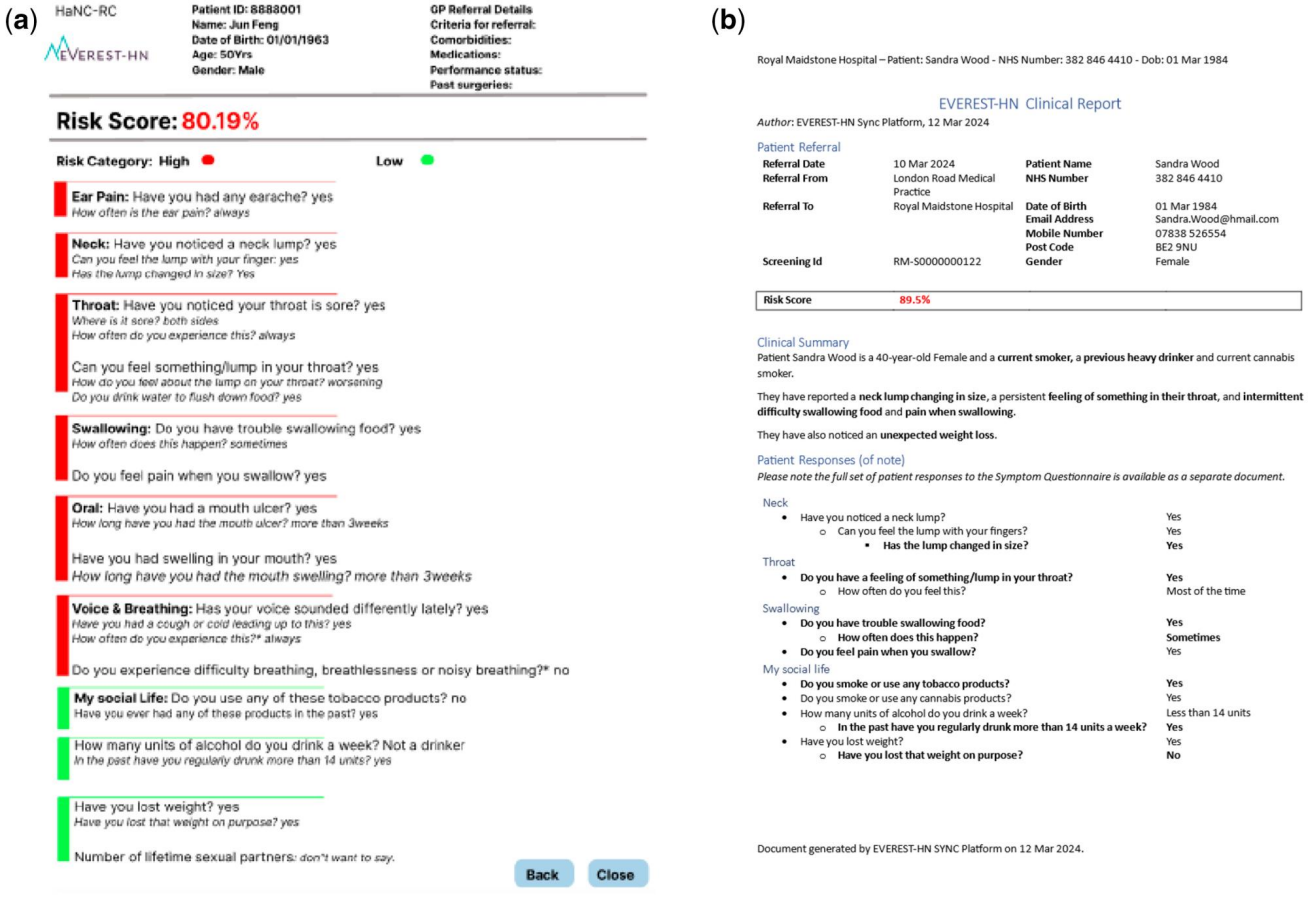
Example of low-fidelity prototype version-2 and the high-fidelity prototype of the Clinician-facing reports.

In Study 1, Site A was used to gather insights from clinicians to define the clinician-facing report. Participants were shown Figures 1a–c generated from patients facing co-design session.^4^ Clinicians provided essential input for the development of the clinician-facing report, generating low-fidelity prototypes (see Figure 1d), which were then refined by the project team (see Figure 2a). In Site B, the low-fidelity prototype developed from ideas from Site A was presented to the participants for validation. Participants provided critical feedback, including strengths and areas for improvement. Site C was used to evaluaate the web-based clinician-facing report and gather suggestions for final improvements.

In Study 2, participants were asked questions designed to: explore the pathway of referrals of suspected HNC received from PCPs, understand potential barriers to integration of the SYNC system, and identify potential solutions to these barriers. See supplementary file 1 for activities that guided the data collection in Study 1 and 2.

### Analysis

The notes from Study 1 and transcripts generated from Study 2 were subjected to thematic analysis, a qualitative method used to identify, analyse, and report patterns (themes) within the data.^10^ While for Study 1 an iterative approach was taken to data collection and analysis, so that the results from each focus group could be fed into the subsequent focus group, for Study 2 all transcripts were analysed together. In both cases, the researcher began by coding and labelling relevant data segments according to the research questions. These codes were then grouped into categories, which represented significant ideas related to the research questions. These categories were further refined and interpreted, with the resulting themes reported in the results section. In preparing this paper, we have followed guidelines for reporting qualitative informatics research1.^11^ To reduce bias, a second researcher, who was present for the first two Study 1 focus groups, reviewed the results of the Study 1 analysis, comparing the results with their recollection of the discussions, and reviewed the transcripts from Study 2 to identify any additional themes.

### Ethics

The research protocol was reviewed by the Committee for Clinical Research at the Royal Marsden (CCR5686) and approval to conduct the research was obtained from the UK Health Research Authority (IRAS 315419). In addition to written consent, verbal consent was taken at the beginning of each session to ensure that participation was voluntary.

## Results

### Study 1—Clinician-facing report

#### Site A—essential features required in the clinician-facing report

Participants were shown the low-fidelity prototype developed during the co-design of the patient-facing questionnaire.^4^ They were also informed about the system dashboard which can track patients’ engagement with the questionnaire. This prototype contained all the HNC questions that the patients would fill when reporting their symptoms. After studying the questionnaire (Figures 1a and b) and the summary page (Figure 1c) that the patient sees once they have completed the questionnaire, the participants made the following recommendations for the design of the clinician-facing report:

- Highlight questions indicating high-risk symptoms in red, and green for low risk to improve decision-making speed and accuracy for clinicians. Participants believed that this visual cue would help to catch their attention and make the report easier to interpret. One participant said *PID001: “It will draw the attention of clinicians’ to key areas of interest.”*
- Incorporate relevant patient details, such as comorbidities, current medications, and performance status, to provide a comprehensive view of the patient’s status. One participant mentioned *PID005: “It would be helpful to include additional patient details like comorbidities to give full picture of the patient’s condition.”* Such information could help to inform decisions regarding investigations and treatment.
- Display the risk score prominently in the report to ensure it is easily noticeable to clinicians.

Participants were enthusiastic about the proposed system and the idea that the dashboard would provide more information about whether patients had completed the questionnaire. They believed that the proposed system would help to make their job easier and the basis for their decisions more transparent.

#### Site B—does the clinician-facing report contain all the information required to decide about patients with HNC symptoms?

The low-fidelity prototype generated from Site A participants’ recommendations (Figure 1d) was further refined with the suggestions from the project team (Figure 2a). Figure 2a was shown to participants in Site B. Participants expressed confidence that the prototype included all the essential questions and necessary information for HNC diagnosis and would be valuable for decision-making. However, they also identified other information that would be beneficial. Specifically, participants felt that information about patients’ past cancer history would be informative at this stage. Additionally, participants identified a couple of areas where they felt further clarity was needed:

- The risk score presented as a percentage in the report. Participants asked for further clarification to show whether the number reflects the likelihood of cancer by providing defined risk categories or explanatory notes. One participant explained *PI001: “I have no clue if this statistic is showing the likelihood of high or low risk cancer. We need more clarifications.”*
- Questions relating to tobacco use. Participants wanted more information where patients reported being former tobacco users. They emphasised the importance of specifying the duration or time frame during which the patient was a smoker.

Some of the participants’ comments conflicted with views expressed by participants in site A. It was felt that performance status was unnecessary at this stage of diagnosis. They were also less enthusiastic about the idea of the dashboard, with comments apparently linked to a reluctance to access a system additional to what they already use.

#### Site C: Exploring the clinician-facing report and feedback for system improvement

Participants were shown the low-fidelity prototype (see Figure 2a) and high-fidelity prototype developed from the idea from Site A and validated in Site B (see Figure 2b). Participants preferred the display features of the report of the high-fidelity prototype as shown in Figure 2b on the basis that it was the format of the report of what they were familiar with. The high-fidelity prototype was designed to show only positive response with the risk score. Participants went through the symptom questions as shown in Figure 2a and provided the following feedback:

- **Ear pain:** Participants did not consider the follow-up question about ear pain, “How often is the ear pain”? relevant. Instead, they suggested including a question about whether the ear pain is unilateral or bilateral to provide more clinical information. One participant said, *PI004: “Would you scrap the, how often is the pain underneath, I don’t think that’s helpful.”*
- **Neck:** The main question was, “Have you noticed a neck lump? with a follow-up question, “Can you feel the lump with your fingers?” Participants were satisfied with how this question was asked because they considered that the question would distinguish between those patients who have a lump on the neck and those patients who have a feeling of a lump internally. *PI004: “I like this question because that distinguishes the patient who has a feeling of a lump internally.”* Another follow-up question was “Has the lump changed in size? Participants considered this question unclear and therefore would not support clinical decision-making, as it could suggest that the lump has got smaller. As one participant explained, *PI002: “This needs to be clarified because it could be a positive thing because the size got smaller, or it could be a very negative thing because it got a lot bigger.”*
- **Cancer history:** Participants considered that patients who had a previous cancer, for example lung cancer, might be at higher risk for HNC. Participants expressed that, even if a patient had a low-risk score, if they had previously had cancer, they would prioritise seeing that patient. Thus, prior knowledge of patients’ cancer history could lead to faster triaging and prioritisation for clinical evaluation. However, participants were clear that the inclusion of a question about previous cancers should be evidence-based, ie if the evidence does not indicate a correlation between previous cancers and the risk of HNC, they would recommend excluding such questions from the system to avoid unnecessary complexity and irrelevant information.

Participants considered that the report included all the critical information required to make informed clinical decisions for patients reporting HNC symptoms. The report displayed all the positive symptoms reported by the patients. One participant said *PID001: “For purely triaging, I would say that the report contains everything that is necessary.”* However, some participants expressed concern that junior doctors or clinicians less familiar with HNC might also benefit from seeing the negative responses. To address this, participants proposed the inclusion of a clickable button that would expand the report to display negative responses as well. Participant *PI004 said: “I think if somebody else was doing a head and neck clinic, for instance someone who didn’t necessarily do head and neck all the time, it probably would be useful to see the negatives as well.”*

Participants also provided the following feedback:

- The overall structure of the report was appreciated for its simplicity. “*PID001: “I think the layout is good and easy to read.”* They like the fact that it highlighted positive symptoms, which contrasts with the current referral forms that is difficult to read due to a lack of emphasis on key details. Participants also compared it with the Excel-based version of the risk stratification tool that they currently use, with one participant commenting *PID001: “the new system would make work easier because somebody would have to fill in the risk score in the previous referrals but with the new system, it would’ve come with it already.”*
- Participants suggested enhancing the visibility of the risk score by increasing its font size and using colour, such as red, or green, to make it more prominent.

### Study 2—the implementation pathway for the SYNC system

During the workshops at each site, the lead researcher introduced the SYNC system including its dashboard features, which tracks patient engagement with the questionnaire system. Participants from each site then shared insights into their current referral pathways for cancer management. The discussions aimed to map out the existing processes and explore how the SYNC system could be integrated to enhance the management of cancer referrals across different hospitals. Table 3, below, provides a summary of the triage process at each site. The implementation of the SYNC system was discussed during workshop sessions at three distinct sites. The aim was to evaluate the feasibility of integrating the SYNC system into existing hospital workflows.

**Table 3.** Triage process across sites

Site	Triaging process
Site A	The outpatient department or booking office is responsible for triaging patients. The triage process is primarily manual, with a clear distinction made between mouth-related and non-mouth-related cases. The oral and dental cases are referred to the maxillofacial (Maxfax) department, while non-oral issues are directed to the ENT department. One participant said, *PID003: “The oral and mouth stuff goes to MaxFac and non-mouth stuff goes to ENT.”*
Site B	Pathway managers review the Appointment Slot Issue (ASI) list daily, which includes patients awaiting triage. The pathway managers notify the relevant departments, eg, the ENT, maxillofacial, or thyroid clinic, via email about the cases that require triaging. The consultants in each department then review the cases and ensure that the referrals are directed to the correct clinic. The departments provide feedback to the pathway managers on whether the patient should remain in their clinic or be redirected to another department. Based on this information, the pathway managers book the necessary appointments or reassign the referral as appropriate. *PID002: “We make the departments aware that these are patients that need to be triaged.”* In Site B whilst the electronic referral documents are uploaded directly, the dental referrals require manual update.
Site C	This site has a more structured approach to triage HNC referrals. They receive a two-page referral form completed by the PCPs. This form includes patient information and details about the suspected cancer symptoms. Once the referral is received, the patient navigator will review the referral letter and complete an additional form (a triage system implemented during the COVID-19 pandemic). This triage form goes beyond simple binary tick boxes and helps to categorise patients as either high or low risk. The initial risk assessment is used to identify the department where the patients need to be seen and then the completed form is sent to booking clerks, who are responsible for scheduling the patient’s appointment. High risk cases are scheduled earlier in the day so that diagnostic procedures (eg, ultrasound or biopsy) can be performed on the patients the same day. Lower-risk cases, such as hoarse voices, are scheduled later in the day as they are less likely to require immediate diagnostic intervention.

#### The triage process across sites

In the sites visited, HNC referrals primarily come from PCPs and some from dentists. Typically, referrals are triaged by administrative or support staff, sometimes with the support of the Head and Neck Cancer Risk Caculator (HaNC-RC). Subsequently they direct referrals to the appropriate team, such as ENT or Oral and Maxillofacial surgery (MaxFac). In line with national targets, patients with an urgent referral should be seen within two weeks and are seen by either HNC consultants or general ENT specialists. For patients with concerning symptoms, such as a history of cancer, a HNC consultant is responsible for their care. If a referral lacks sufficient clinical detail, the referral may be sent back to the PCP for more information (eg, vague details like simply ticking “hoarse voice” are insufficient for accurate triaging). While delays due to incomplete PCP referrals are rare, they do occur. MaxFac referrals come from both PCPs and dentists. These referrals are reviewed by two HNC consultants to confirm their validity. Dental referrals often include images, and in some cases, patients may receive advice based solely on the provided images.

#### The feasibility of integrating the referral into the SYNC system

Discussions focused on operational feasibility from the perspective of cancer management staff. Participants deliberated on how the referral process, currently managed via the e-Referral Service (ERS), could be adapted to include the SYNC platform. They considered the potential of virtually downloading referral documents from the ERS and then uploading them to the SYNC system at the points of administrative workflow, for instance when appointments are manually allocated, letters are sent to patients via text, or virtual documents are printed to hospital systems. While the participants acknowledged that uploading referral documents to SYNC seemed technically feasible, several complexities were raised:

- Information governance (IG) considerations: since the SYNC system operates outside of the traditional hospital IT infrastructure, there were questions about whether integrating the new system would comply with current IG policies. Participants advocated for consulting the hospital’s IG team to confirm that patient data could be securely transferred into the SYNC system. Some participants mentioned *Site A PID004: “IG would be the major determinants of whether or not we can do this new system.” Site B PID002: “if we are virtually printing the referral, then it would be no different to uploading the questionnaire.” Site C PID001: “Before we can upload any document into the SYNC, involving information governance is important”*
- Automation vs manual upload: there was debate over whether the process should be automated by the IT department or managed manually by administrative staff. Some participants argued that automating the process would minimise human error, while others suggested that the manual method, virtually printing referral forms and sending them to SYNC should ensure compliance with IG requirements. *Site C PID001: “for any new system coming in, it would have to follow the new implementation rules via IT and data transfer protocols.”*
- **Motivating and engaging staff for long-term involvement** 
  Participants from the three sites had differing views on how best to motivate staff to ensure uptake continued use of the SYNC system: Site A participants advocated for short instructional videos to educate staff on how to use the system. They believed that for SYNC to be widely adopted, it must clearly demonstrate its ability to improve clinical practices. Site B participants believed that no additional training or instructional videos were necessary. Rather, they argued that the system should be designed to be as simple as possible and should eliminate the need for extensive onboarding. One participant stated, *Site B PID004: “No need for clinician training or instructional videos, the system should be as simple as possible.”* Site C participants emphasised the importance of short videos as well as training sessions, especially for administrative and support staff. They felt that clear guidance would be essential for ensuring smooth adoption and prevention of potential errors during the implementation phase. One participant remarked, *Site C PID001: “It should be helpful if someone sit with us and show us how things are done before it is up and running, to make sure we weren’t making any mistakes.”*

#### Challenges and barriers to SYNC system implementation

The following challenges were identified: Participants from outpatient departments expressed concern over the lack of access to patients’ email addresses. This would hinder the ability to send out the digital questionnaires to patients. However, the researcher reassured participants that SYNC would have the capacity to also send links via text message to patients’ phones. Another concern raised was the voluntary nature of the symptom questionnaire and whether patients would complete it. The researcher explained that the SYNC system would include a dashboard feature to track whether patients had received, started, or completed the questionnaire. The system also has the capacity to send reminders to patients on three occasions. Participants highlighted the emotional impact on patients when informed by their GP that they were being referred for a potential cancer diagnosis. The focus on the word “cancer” could cause patients to overlook critical information, such as instructions about completing the questionnaire. One suggestion was for GPs to immediately provide the link to the questionnaire during the consultation to reduce the chance of confusion or non-compliance.

In the context of increasing awareness around cybersecurity threats, participants raised concerns that patients may be reluctant to click on links sent via text, fearing they might be phishing attempts or scams. This fear could potentially reduce patient engagement with the digital questionnaires. Participants recommended combating this apprehension by providing information within the text or email that would provide assurance of legitimacy or having GPs inform patients that they would receive the questionnaire. *Site A PID006: “It is potentially a problem as more and more people are aware of not clicking on links within messages.”* It was also noted that there was a mismatch in decision-making timelines: the proposed system would give patients 72 hours to complete the questionnaire. However, clinicians in Site A currently need to make triaging decisions within 48 hours, which presents a significant conflict. Participants called for this timing issue to be resolved to ensure that clinical workflows are not disrupted.

## Discussion

The research aimed to design a clinician-facing report generated from a digital questionnaire to enhance the care management of suspected HNC patients and assess the integration of the SYNC system into clinical workflows. The research gathered insights from clinical and non-clinical staff about the potential benefits and challenges of the system. For the clinician-facing report design, participants prioritized a clear, report format with color-coded risk scores (eg, green/red) and access to patients’ cancer history was considered useful for decision-making and prioritisation. Opinions varied on training needs and the value of the dashboard. Site A expressed a positive view of the system, suggesting that it would be a helpful tool, particularly for more junior staff, provided that appropriate training is offered. In contrast, Site B questioned the utility of the dashboard, noting that clinicians often lack the time to engage with additional system features. They emphasised the importance of keeping the system as simple and streamlined as possible, expressing concern that additional training requirements might deter usage. These differing perspectives may reflect underlying contextual factors, such as variations in clinical workload, digital confidence, or institutional support for training. Site A may have greater flexibility in staff time allocation or a culture more open to digital innovation, whereas Site B may face tighter constraints on time and capacity, prompting a preference for minimal disruption to existing workflows. This suggests there will be varying levels of adoption based on local workflows.

For the implementation pathways, concerns included the feasibility of integrating referral documents while ensuring IG compliance. To manage these concerns, a comprehensive IG framework was embedded from the outset. This included the completion of a Data Protection Impact Assessment (DPIA), detailing the lawful basis for processing health data, data minimisation practices, secure data storage protocols, and retention policies in line with UK GDPR and the Data Protection Act 2018. Additionally, the SYNC platform has been assessed using the NHS Digital Technology Assessment Criteria (DTAC), which explicitly covers information governance as a core domain. To strengthen IG compliance, external consultancy support was engaged to ensure that all aspects of data protection, cybersecurity, and NHS-specific governance requirements were met. To further support clinical acceptability and regulatory readiness, approval from the Medicines and Healthcare products Regulatory Agency (MHRA) has been sought for the evaluation phase. In practice, participants will be provided with clear information about data use, storage, and rights at the point of engagement, and consent processes will include reassurances about how personal data are protected. These measures are intended to enhance transparency, build user trust, and support the system’s acceptability and scalability within hospital settings.

The process of designing the clinician-facing report and exploring implementation strategies for the SYNC system provided researchers with valuable insights into the development and integration of digital healthcare tools. Table 4 illustrates the lessons learnt.

**Table 4.** Lessons learnt from the studies

Lesson learnt	Description
Stakeholder Engagement	Engaging a wide range of stakeholders in the design process ensures systems meet real user needs and account for different stages of the patient pathway and implementation challenges.
Iterative Feedback	Continuous feedback from users helps refine systems to suit real-world workflows. A single approach won’t fit all healthcare settings due to differing needs and priorities. This supports findings that ease of use is key to adoption.
Usability vs Clinical Support	Systems like SYNC must be easy to use while also supporting complex clinical decisions. The Technology Acceptance Model (TAM) highlights those emotional reactions, such as frustration from poor workflow, affect continued use.
Training Flexibility	Training requirements vary—from no support to guidance from senior consultants. Health technologies should provide adaptable onboarding options tailored to user roles and context.
Information Governance (IG) Integration	IG protocols can be barriers to implementation. Engaging IG teams early and maintaining ongoing communication is crucial for compliance and smooth system integration.
Patient Trust and Communication	Patients may mistrust digital systems due to online scams. Introducing systems through trusted sources like GPs and using clear, credible messaging (eg, via SMS/email) can build trust. Concerns around data privacy remain a significant barrier to digital health adoption.^12^^,^^13^

### Study limitations

The number of clinicians and healthcare staff involved in the study was relatively small and limited to specific sites. Additionally, clinicians or administrative staff who were more interested in digital health solutions may have been more likely to participate, potentially skewing the findings. This may limit the generalisability of the findings across different healthcare settings. The diverse opinions and needs expressed across sites might not represent all possible viewpoints, particularly from clinicians in other hospitals that use different referral or triaging systems. Another limitation is that feedback was based on initial impressions rather than extended use. However, a planned randomised controlled trial of the SYNC system will provide the opportunity to gather feedback from a greater number of clinicians and will provide insight into use over the longer term and whether this translates into improvements in patient care.

The absence of IT staff in site B and C (at site A, IT staff joined the beginning of the meeting online, to inform us of lack of capacity) limited the depth of technical discussions to other cancer management staff.

### Conclusion

This study highlighted the critical role of involvement of a range of staff in the design and integration of digital tools for HNC care. The research identified key factors likely to influence successful adoption of the SYNC system. These include the clarity of information presented, the perceived usefulness of the tool in improving workflow efficiency, and the ease of integrating new technology into daily practices. The results suggest that, when a healthcare technology device is designed with user feedback and operational realities in mind, digital health tools like SYNC have the potential to enhance patient care and decision-making. However, challenges such as ensuring consistent usage, minimising disruption to workflows, and providing adequate training must be addressed to facilitate successful integration. While the study offers valuable insights into the design and potential implementation of the SYNC system, the findings are based on data from three hospital sites within a specific regional context in the United Kingdom. As such, the extent to which these results can be generalised to other settings may be limited. Differences in institutional priorities, digital infrastructure, staffing levels, and patient populations could influence the acceptability and integration of the system elsewhere. However, the study highlights cross-cutting themes such as the need for workflow compatibility, clear information governance processes, and inclusive design that are likely relevant across various secondary care environments.

## Supplementary Material

ooaf130_Supplementary_Data

## Data Availability

The data underlying this article will be shared on reasonable request to the corresponding author
