# Supplementary material for: Co-design of clinician-facing report and implementation pathway for a digital questionnaire for reporting head and neck cancer symptoms
Source: JAMIA Open. 2025 Nov 12;8(6):ooaf130. doi: 10.1093/jamiaopen/ooaf130 (PMC12612671; doi:10.1093/jamiaopen/ooaf130)
Supplement: ooaf130_Supplementary_Data [file ooaf130_supplementary_data.zip › Supplementary file 1.docx]

**Supplementary file 1: Activities that guided study 1 and 2**

**Study 1**

**Site A:** Activity 1: Understanding EVEREST-HN project and prototype assessment:

- The researchers briefed participants about the EVEREST-HN project and its objectives and then displayed the low-fidelity prototype featuring patient symptom questionnaire and summary page see Figs. 1a, b and c.

Activity 2: Design Assessment

- What are the essential features and content required in the PDF report following patient questionnaire completion? This question was asked to gather insights from participants regarding their expectations and preferences for the content and features of the clinician-facing report. The researchers want to understand how participants believe the report should be structured and presented to meet the needs of clinicians.
- Could you try to give an idea to show the layout of the report and explain the reason behind their design choices. This task was given to participants to help researchers gain a clear understanding of the desired structure and presentation of the report from the perspective of the clinicians.

**Site B:** The researchers displayed the low-fidelity prototype generated from the ideas in site A (see Fig. 1d and 2a). Then, the following questions guided the activities in site B.

- Looking at the clinician-facing report prototype, do you think that it contained all the information required to take decisions about patients with HNC symptoms? This question was posed to identify any gaps in the prototype by comparing it against the expectations and needs of clinicians. The aim was to determine if there were additional elements or information that might have been overlooked and to ensure that the report is comprehensive enough to support clinical decision-making.
- What do you think is needed for the intervention to work? This question was asked to identify potential bottlenecks or challenges that could hinder the successful implementation and usage of the SYNC system by clinicians.

**Site C:** The researcher displayed the idea of the high-fidelity prototype generated from the ideas from site A and B and allowed the participants to explore the prototype. The following task guided the data collection in site C.

Activity 1: Understanding EVEREST-HN project and prototype assessment, the researcher carried out the following:

- Brief participants about the EVEREST-HN project and its objectives.
- Display the low-fidelity prototype developed from the previous sites.
- Display the paper-based idea of the web-based SYNC system.
- Allow participants to make some inputs.

Activity 2: Improving the clinician-facing report.

- Does the report include all the critical information you need to make informed clinical decisions? If not, what additional information would be useful. This question was asked to ensure that the SYNC system report provides clinicians with all necessary information to make informed decisions before the patient arrives at the hospital.
- Is the information in the report presented in a clear and understandable way? Are there any sections that are confusing or need further explanation? This question was intended to ensure that the report’s content is accessible and easily understood by all members of the clinical team.
- Would you suggest any changes to the layout or organisation of the report to make it more user-friendly? This question sought to verify that the layout produced by the high-fidelity prototype was user-friendly and acceptable to the clinical team.
- How do you think this report will integrate into your existing clinical workflow? Are there any adjustments that could improve its fit within your daily practice? This question was asked to identify any potential adjustments that may be needed to better integrate the report into the clinical workflow, with the intention of communicating these adjustments to the broader project team for further refinement.

**Study 2** - The implementation pathway: participants in each focus group were asked to discuss the current referral processes and the potential challenges that may hinder the integration of the SYNC system into their clinical practice. The following research questions guided study 2.

- What is the process of triaging across sites?
- What is the feasibility of integrating the referral into the SYNC system?
- How do you motivate and engage staff for long-term involvement?
- What are the challenges or barriers to the SYNC system implementation?
